# Intermittent claudication services in England: insights from a freedom of information request

**DOI:** 10.1186/s12913-026-14215-9

**Published:** 2026-02-23

**Authors:** Smaragda Lampridou, Layla Bolton Saghdaoui, Chloe Boobier, Gaby Judah, Mary Wells, Alun Huw Davies

**Affiliations:** 1 Vascular Surgery Department, Imperial College Healthcare NHS Trust, London, UK; 2 Faculty of Medicine, Department of Surgery & Cancer, Imperial College London, London, UK; 3 Nursing Directorate, Guys and St Thomas NHS Foundation Trust, London, UK

**Keywords:** Peripheral artery disease, Intermittent claudication, Supervised exercise therapy, Vascular services, Freedom of information, Healthcare disparities

## Abstract

**Background:**

Peripheral arterial disease (PAD), presenting as intermittent claudication (IC), affects 20% of adults over 60 years of age in the UK. Claudication reduces patients’ mobility, quality of life, and increases their risk of cardiovascular mortality. National guidelines recommend a holistic, multidisciplinary approach including supervised exercise therapy (SET), pharmacotherapy, and lifestyle modifications. However, clinicians raise concerns about inconsistent service provision across NHS England. Freedom of information requests (FOIR) have become an important tool for researchers to access government information and examine practices. This study aimed to explore the availability and structure of services for the management of patients with IC in England.

**Methods:**

A FOIR was distributed to NHS Trusts in England in January 2025. The request included questions about IC clinics, SET availability, staffing, access to lifestyle and psychological support, and the referral process to IC services. The questionnaire was piloted with vascular specialists and was refined before dissemination. The data were analysed using descriptive statistics in SPSS (v29).

**Results:**

Of the 138 Trusts contacted, 124 (89.9%) responded, with 82 (66.2%) providing vascular services. Fewer than half (46.3%) offered dedicated claudication clinics, and only 29.3% provided structured SET programmes. Yet of these SET programmes, only 12.5% of were delivered in line with national guidelines. Most clinics were nurse-led, whereas most SET programmes were physiotherapy-led. Lifestyle support was limited: only 22% provided smoking cessation services internally, 25.6% offered dietetic support within the Trust, and just one Trust reported access to psychological services for PAD patients. Most Trusts (94%) used an electronic referral form, whereas only 34% had a specific vascular proforma.

**Conclusion:**

This FOIR highlights substantial regional variation and gaps in claudication service provision across England. Many Trusts do not consistently deliver care aligned with national clinical guidelines, particularly in offering SET and integrated lifestyle care. Future research should focus on bridging the gap between policy and practice, expanding access to evidence-based interventions, and ensuring equitable, holistic care pathways for patients with IC.

**Supplementary Information:**

The online version contains supplementary material available at 10.1186/s12913-026-14215-9.

## Background

Peripheral arterial disease (PAD) affects approximately 20% of individuals aged 60 and over in the United Kingdom (UK) [1, 2]. Many patients present with intermittent claudication (IC), pain and cramping in the legs caused by inadequate blood flow during physical activity [1, 3]. Claudication not only impacts patients’ physical mobility but also diminishes their quality of life and increases their risk of cardiovascular and lower limb events [1–4]. National and international clinical guidelines stress the importance of cardiovascular risk reduction in patients with IC [5, 6], through pharmacotherapy and lifestyle changes. Guideline-recommended pharmacotherapy includes antiplatelet or antithrombotic medications, lipid-lowering and antihypertensive agents (for patients with hypertension). Recommended lifestyle modifications are smoking cessation, healthy diet, weight management, regular exercise, and attending a supervised exercise therapy programme (SET) [5, 6].

For patients with IC, timely diagnosis and access to appropriate care and disease management, including vascular specialist clinics and SET programmes, are crucial for improved outcomes. Despite this, research has shown that service provision across the UK remains inconsistent, with limited availability of SET programmes [7–9]. Previous research on claudication services in England has solely focused on SET provision, without fully addressing other key aspects of care, such as structured smoking cessation support or the organisation and setup of dedicated claudication clinics. A more comprehensive understanding of the breadth and quality of these services is therefore required.

Freedom of information requests (FOIR) have emerged as a valuable tool for researchers to access detailed, system-level data in healthcare [13, 14]. In the context of the UK healthcare system, which is complex and continually evolving, FOIRs offer an invaluable method for acquiring a clear picture of available healthcare provisions, including specialised services for specific conditions, such as claudication.

This study aimed to explore the availability and structure of services for the management of patients with IC in England. The specific objectives were to characterise the structure and delivery of IC clinics and to examine the availability of SET, lifestyle and psychological support services. Additionally, the referral processes to IC clinics was assessed. By understanding the current service provision, this work can help guide targeted efforts to enhance care equity, service planning, and patient outcomes within vascular care.

## Methods

### Study design

A pragmatic, descriptive mapping exercise, utilising a FOIR approach, was conducted to explore the set-up of IC services across England. To address the study aim, the FOIR consisted of a structured questionnaire on various aspects of service provision, informed by national guidelines [5] and previous studies on vascular SET services [8]. The questionnaire was specifically developed for this study, and although not previously validated or published, it followed questionnaire formatting guidance [10, 11]. It includes 22 questions, both multiple-choice and open-ended. All questions included an “Other” option with space to provide a written response, in case none of the multiple choices matched their organisation’s service. A definition of vascular services was also provided alongside the questionnaire [12, 13]. To ensure clarity and relevance, the questionnaire was piloted with a small group of vascular specialists (in addition to the vascular authors involved in the project) and refined accordingly before dissemination.

The questionnaire (see Supplementary Material 1) covered the following areas:

- The existence and structure of IC management services within institutions.
- The range of healthcare professionals involved in IC care (vascular surgeons, vascular nurses, physiotherapists, etc.)
- The extent of SET availability, including whether it is delivered in hospital-based, community-based, or virtual settings.
- The inclusion of smoking cessation programmes, dietary counselling, and psychological support for patients with IC.
- The referral process to IC services.

### Setting

The National Health Service (NHS) England, established in 1948, is founded on the principles of universal access, equity, and care on the basis of clinical needs rather than the ability to pay [14]. Funded primarily through general taxation, NHS England provides comprehensive healthcare services across primary, secondary, and tertiary care [14]. Increasing demand, rising costs, and staff shortages have led to major reforms. These changes aim to improve efficiency and integration, with greater emphasis on local planning and coordination between NHS England Trusts, Integrated Care Boards (ICBs), and community services [15].

Vascular services in England are largely delivered within this framework by specialist teams based in acute NHS England Trusts, often supported by regional vascular networks, aiming to coordinate care, centralise complex procedures, and improve outcomes [12, 13]. These services include the diagnosis, medical management, and surgical treatment of conditions affecting the arterial, venous, and lymphatic systems. Patients with IC may be reviewed in either a specialised IC clinic or a generic vascular clinic, depending on the set-up of each organisation [12, 13]. In recent years, vascular services have undergone significant re-organisation to consolidate specialist expertise and resources into fewer high-volume centres, supported by evidence suggesting improved patient outcomes through centralisation [12, 13]. Despite progress, challenges remain, including variations in service provision, workforce shortages, and unequal access to care across regions.

### Participants and data collection

The FOIR was sent to NHS England Trusts on the 21st of January 2025, requesting information about services provided for patients with IC. Trusts solely providing mental health, cancer, women’s or paediatric services were excluded from the FOIR.

The request was sent via email or submitted via Trust-specific electronic forms to the designated FOIR teams at NHS England Trusts, following the official procedures outlined in the Freedom of Information Act 2000 [16]. Trusts that provided incomplete responses were contacted to obtain the missing information. Non-responding institutions were sent reminders after the statutory 20-working-day period, and thereafter at four-week intervals, to encourage participation [16]. Where possible, additional contact was made with key personnel within vascular services. Reminder emails continued up to three times over 12 weeks unless a response was received earlier.

The Freedom of Information Act 2000 enables access to publicly held, non-identifiable information [16]. As no patient-level data were collected, this study did not require ethical approval. The study was conducted and documented following the guidelines outlined in the Strengthening the Reporting of Observational Studies in Epidemiology (STROBE) checklist [17].

### Data analysis

All responses were collected, recorded, and stored in a secure Microsoft Excel spreadsheet for preliminary organisation. The data were then cleaned for consistency. Using the IBM Statistical Package for Social Sciences (SPSS) (v.29) software, descriptive statistics were used to summarise the quantitative data.

## Results

### Responders

From 21 January 2025 to 21 May 2025, 138 Trusts were sent a FOIR. There was an 89.9% response rate, with 124 Trusts submitting a response. At least three reminders were sent to the 14 Trusts that did not respond. Of the 124 Trusts that responded to the FOI request, 12 (9.6%) were primary care Trusts and reported not providing vascular services, and 30 (24.2%) were secondary care Trusts not providing vascular services. Finally, 82 (66.2%) were secondary care Trusts reporting that they provided vascular services.

**Fig. 1:**
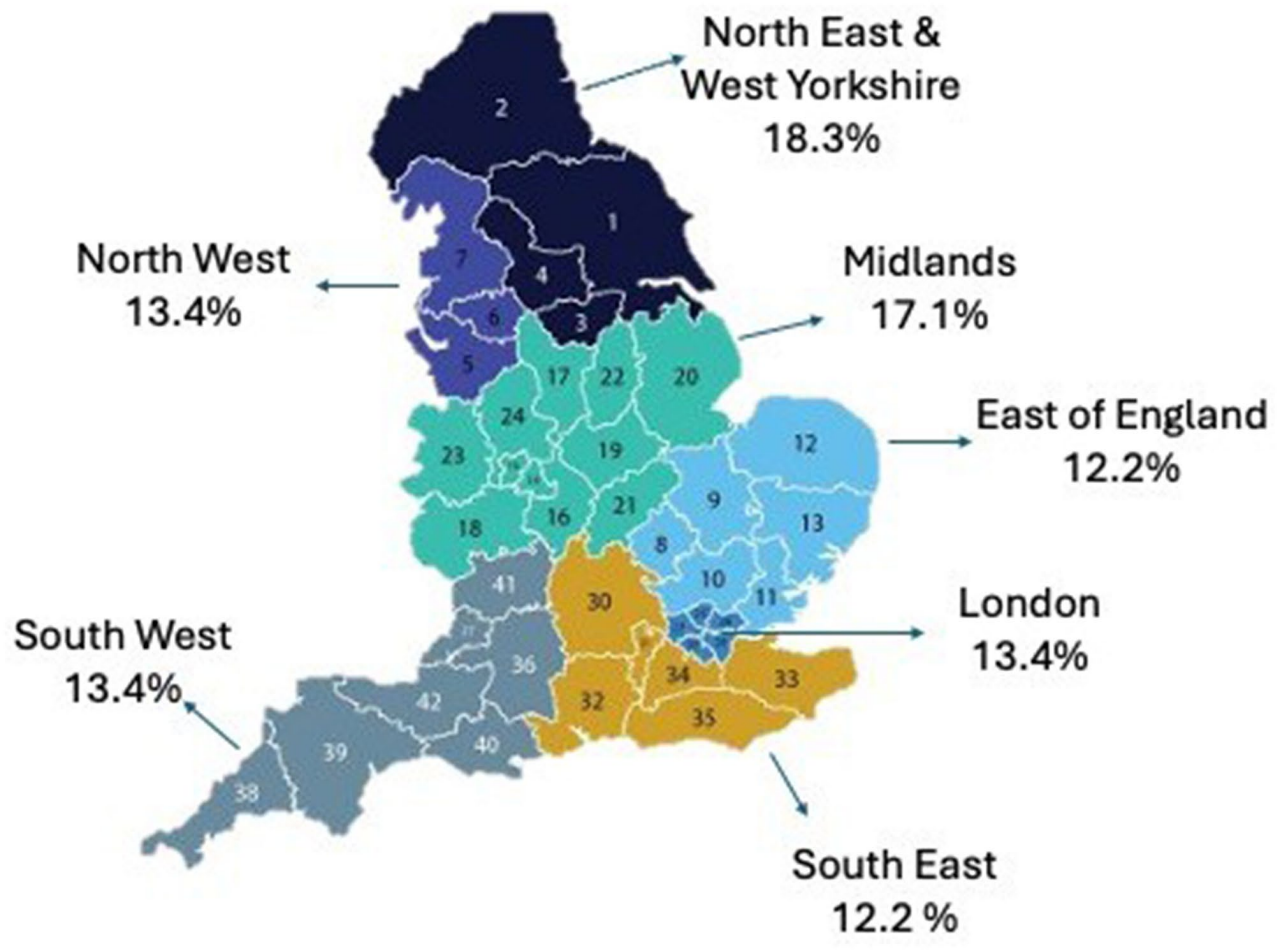
Geographical distribution of the 82 NHS England Trusts with vascular services that responded to the FOIR

The 82 Trusts included in the analysis were distributed across the integrated care systems in England, as shown in Fig. 1. The FOIR demonstrated a broadly even geographical distribution of vascular services, with the North East and West Yorkshire contributing the highest proportion (18.3%).

### Claudication clinics

Of the 82 Trusts that responded to the FOIR and which provide vascular services, 38 (46.3%) reported offering specific claudication clinics. Of these, 19 (50%) were nurse-led, 11 (29%) were jointly run by doctors and nurses, and eight (21%) were jointly run by vascular nurse specialists and allied health professionals (AHP). Regarding services offered, two clinics (5.3%) provided disease counselling, treatment planning, lifestyle management, and verbal exercise advice; 20 clinics (52.6%) offered these services along with diagnostic testing; and 16 clinics (42.1%) provided the full range of services, including diagnostic testing and structured exercise programmes. In terms of clinic frequency, one (2.6%) ran once a month; six (15.8%) every 2–4 weeks; 23 clinics (60.5%) ran weekly; seven (18.4%) three times a week, 1 (2.6%) once a month, and one (2.6%) daily. Follow-up intervals were most commonly determined by clinician decision (24 clinics, 63.2%), while others offered follow-up every 3–6 months (11 clinics, 28.9%), once a year (2 clinics, 5.3%), or had no formal follow-up (1 clinic, 2.6%).

Regarding waiting times for patients with IC, from referral to their first vascular specialist clinic appointment, 47 out of 82 respondents (57.3%) did not provide an answer. Among those who did, 2 (2.4%) said it depended on urgency, 8 (9.8%) reported a waiting time of less than 4 weeks, 14 (17.1%) reported 4–12 weeks, and 11 (13.4%) indicated a waiting time longer than 12 weeks.

Regional variation was evident (see Table 1). South East and London were among the best served, with 70% and 62% of Trusts providing claudication clinics, respectively. North East and Yorkshire also demonstrated relatively higher provision, with 53% of Trusts offering clinics. Service provision was considerably lower in the North West, Midlands, and East of England.

**Table 1:** Regional distribution of claudication clinics and SET across responding trusts

Region	Trusts Responding to the FOIR	Trusts with Vascular Services	Trusts with Claudication Clinics	Trusts with SET
North East & Yorkshire	27	15	8	5
North West	16	10	3	2
Midlands	20	13	3	4
East of England	13	10	3	4
South West	19	11	6	2
South East	12	10	7	3
London	17	13	8	5
Total	124	82	38	24

### Exercise programmes

Of the 82 Trusts responding to the FOIR, 24 (29.3%) reported offering SET programmes and structured exercise advice for patients with IC, whereas the remaining 58 Trusts (70.7%) provided only verbal exercise advice without a formal SET programme. Of these 24 Trusts offering SET, 16 Trusts offered claudication clinics and SET; however, in eight of the Trusts offering SET, patients may have been in generic vascular clinics, as they did not have dedicated claudication clinics.

Among the 24 Trusts delivering a structured exercise intervention (see Table 2), the format of the exercise programme varied as follows: 8 Trusts (33.3%) offered supervised classes exclusively in a hospital or community setting; 12 Trusts (50%) combined supervised classes in hospital or community settings with home-based, unsupervised exercise; 2 Trusts (8.3%) paired supervised classes with an app‐based home programme; 1 Trust (4.2%) combined supervised classes with virtual (home‐based) supervised sessions; and 1 Trust (4.2%) provided an app‐directed exercise programme only. Programme location was predominantly within a secondary care setting: 16 Trusts (66.7%) delivered SET in hospital, 4 Trusts (16.7%) referred patients to a different hospital, 2 (8.3%) referred patients to a primary care setting outside the Trust, and 2 (8.3%) referred to a private setting outside the Trust, specifically in a university and a local charity.

**Table 2:** Characteristics of exercise provision for patients with IC in responding trusts

Characteristic	*n* (%)
**Exercise Format**	
Supervised classes (hospital or community)	8 (33.3)
App-based home exercise only	1 (4.2)
Supervised + home-based unsupervised	12 (50.0)
Supervised + app-based home	2 (8.3)
Supervised + virtual supervised at home	1 (4.2)
**Programme Location**	
Within own Trust	16 (66.7)
Another hospital/Trust	4 (16.7)
Referral to primary care (outside Trust)	2 (8.3)
Referral to private setting	2 (8.3)
**Programme Duration**	
4–8 weeks	7 (29.2%)
12–16 weeks	16 (66.7)
6 months	1 (4.2)
**Session Length (minutes)**	
30–60	17 (70.8)
60–90	6 (25.0)
90	1 (4.2)
**Session Frequency (times/week)**	
Once per week	10 (41.6)
Twice per week	4 (16.7)
One or two times per week	3 (12.5)
> 3 times per week	3 (12.5)
Dependent on clinical decision making	1 (4.2)
Every 2–4 months	1 (4.2)
No response	2 (8.3)
**Staffing**	
Physiotherapist alone	6 (25.0)
Exercise professional alone	5 (20.8)
Physiotherapist + nurse	4 (16.7)
Physiotherapist + nurse + exercise professional	2 (8.3)
Nurse + exercise professional	1 (4.2)
Physiotherapist + physio aide	1 (4.2)
Nurse + sonographer	1 (4.2)
Nurse alone	1 (4.2)
Nurse + vascular scientist	1 (4.2)
Exercise professional + BACPR exercise physiologists	1 (4.2)
Vascular surgeons + vascular nurses + vascular scientists	1 (4.2)

In terms of programme duration, seven Trusts (29.2%) offered programmes lasting 4–8 weeks, 16 Trusts (66.7%) reported that supervised exercise programmes typically lasted 12–16 weeks; and one Trust (4.2%) had a 6-month programme.

The frequency of supervised classes also varied: one Trust (4.2%) based frequency on clinical decision-making; one Trust (4.2%) scheduled classes every 2–4 months; 10 Trusts (41.6%) held sessions once per week; three Trusts (12.5%) met one or two times per week; four Trusts (16.7%) twice weekly; three Trusts (12.5%) held sessions three times per week; and two Trusts (8.3%) did not provide an answer.

Shorter programmes (4–8 weeks) were limited in frequency, with only four delivered once per week, and sessions were typically 30–60 min (5 programmes) or 60–90 min (3 programmes) (see Table 3). Programmes taking place for 12–16 weeks ran once per week (5 programmes), 1–2 times per week (3 programmes), or 2–3 times per week (3 programmes each), with one programme delivered very infrequently (every 2–4 months).

The length of the exercise classes was most commonly 30–60 min, as specified by 17 Trusts (70.8%); six Trusts (25%) ran 60–90–minute classes; and one Trust (4.2%) conducted a 90-minute session.

Regarding compliance with the NICE guidelines (12–16 weeks, two hours) [5], only three out of 24 Trusts (12.5%) were delivered in accordance with national guidance (see Table 3). According to the updated 2024 European Society for Vascular Surgery (ESVS) clinical practice guidelines on exercise therapy for PAD [18], another three of 24 Trusts (12.5%) delivered exercise therapy in accordance with these guidelines.

**Table 3:** Distribution of exercise programmes by duration and format

Programme Duration	Frequency					Length of session (minutes)		
	1/week	1–2/week	2/week	3/week	Every 2–4 months	30–60	60–90	90
4–8 weeks	4*	-	-	-	-	5	3	-
12–16 weeks	5	3	3	3	1	9	3	1
> 16 weeks	-	-	1	-	-	1		-

*The numbers represent the number of exercise programmes that fit the characteristics

Finally, the multidisciplinary composition of the teams delivering SET varied, with physiotherapists being the most common profession running the classes, either alone in 6 hospitals (25%) or together with another professional in 7 hospitals (29.2%) (see Table 2). Four Trusts (16.7%) combined a physiotherapist and a nurse, whilst 2 Trusts (8.3%) had a team of a physiotherapist, a nurse, and an exercise professional.

### Lifestyle and psychological support services

Regarding smoking cessation services, within their own organisation, 18 respondents (22.0%) reported providing smoking cessation support, while three (3.7%) referred to another hospital or Trust outside their organisation. The most common pathway was referral to a primary care setting outside the organisation, reported by 34 respondents (41.5%). Additionally, 5 (6.1%) referred patients to private services, and 16 (19.5%) used a combination of hospital services and primary care referrals. Two out of 82 Trusts (2.4%) reported offering cessation advice only, two (2.4%) did not formally refer patients, and two (2.4%) did not provide an answer to the question.

For dietetic services, 21 Trusts (25.6%) reported providing services within their own organisation, while 2 (2.4%) referred to another hospital or Trust outside their organisation. Referral to a primary care setting was offered by 21 Trusts (25.6%), two (2.4%) referred to a private setting, and six (7.3%) reported offering both hospital support and referral to primary care. Two (2.4%) offered advice only, and 23 (28.0%) stated they did not offer any referral or dietetic service. Seven out of 82 Trusts (8.5%) did not provide an answer to the question.

Psychological support services for patients with claudication were notably limited, with only one Trust (1.2%) reporting that they provided such services.

### Referral process for IC services

Most of the responding organisations (94%) reported the use of electronic referral systems for patients with IC. A small minority relied exclusively on alternative methods, including secure email (*n* = 3) or paper-based referral (*n* = 1), while one organisation did not provide an answer to this question. When asked whether a standardised referral proforma was in use, only 28 organisations (34%) reported having an established template, with an additional two (2.4%) indicating that a specific vascular referral proforma was under development. The remaining majority (64%) did not use a standard proforma.

## Discussion

### Main findings

This FOIR was the first to examine the availability and structure of claudication services across NHS England, including the provision of lifestyle support and SET. The findings reveal marked disparities in care provision, despite NHS England’s commitment to universal healthcare access. Fewer than half of centres offer dedicated claudication clinics, less than a third provide SET programmes, and even fewer provide integrated support services such as smoking cessation, dietary or psychological counselling. These service gaps suggest that many patients with IC may not receive guideline-recommended, holistic care, raising concerns about equity, consistency, and long-term outcomes. Although existing literature on claudication services in England is limited, the results of this FOIR align with earlier studies highlighting poor availability of SET, and further underscore the systemic variation in vascular service delivery across the country.

Although we have moved on from the pandemic era, where vascular services have provided mainly emergency procedures [19], the limited availability of claudication services and SET, despite their strong evidence base, suggests a gap between clinical guidelines and real-world practice. A previous retrospective cohort study evaluated the effects of the 2012 NICE guideline on the management of PAD in NHS England, using Hospital Episode Statistics (HES) data from 2009 to 2019 [20]. The study highlighted that the introduction of the NICE guidelines was linked to a reduction in hospital admissions for revascularisation, particularly among patients with moderate IC [20]. Unfortunately, there are no data on the provision of medical management and lifestyle services for patients with IC, since the implementation of the guidelines.

Our FOIR revealed that all claudication clinics and most SET programmes are delivered exclusively within secondary care, limiting accessibility for many patients, particularly those in rural or underserved areas. To ensure equitable access, services should be available in both primary and secondary care settings. Although such a care pathway was proposed by NHS Scotland in 2024 [21], NHS England has yet to approach a similar model. The recently published Commissioning for Quality and Innovation (CQUIN) for the best clinical practice management of PAD, stresses the importance of cardiovascular risk factor management, but offers little guidance on how to optimally deliver such services to meet these goals [9]. Models from other chronic disease pathways, such as cardiac rehabilitation and oncology, have demonstrated that nurse-led clinics can improve patient outcomes, enhance quality of life, and provide cost-effective care through personalised education, regular monitoring, and psychosocial support [22, 23]. Similarly, a recent study assessing the impact of a nurse-led IC clinic revealed that such clinics can streamline vascular services by accurately diagnosing and managing PAD, therefore reducing demand on doctor-led clinics and prioritising specialist input for patients with more urgent surgical needs [24]. Given these proven benefits, nurse-led clinics should be positioned as a cornerstone of care for patients with IC, particularly during early management. These clinics could support cardiovascular risk reduction and lifestyle modification, provide early education, and enable timely referral to SET programmes or other vascular specialists. To deliver a more optimal service, future care models should also include closer integration with primary care, standardised referral pathways, and robust data collection on outcomes to inform continuous improvement. Such steps are essential for reducing regional variation and ensuring that all patients with IC receive timely and comprehensive care.

With regards to SET, our FOIR found a wide variation in programme format, setting, duration, session length, frequency, and staffing. Although SET is recommended for all patients with IC, limited NHS England commissioning [25] means that many Trusts do not offer it at all. Since the Covid-19 pandemic, there has been a rise in digital interventions aimed at improving SET availability and adherence. The use of smartphones and wearable devices enables real-time monitoring, personalised feedback, and accessibility without geographic constraints [26]. A systematic review has shown that mobile health (mHealth) for PAD is associated with improved adherence and effectiveness [27]. However, only four Trusts in our FOIR reported utilising digital health approaches to SET. While limited use of technology may reflect assumptions about the frailty, high comorbidity and digital literacy in the PAD population, digital options should still be made available for more technologically able patients and those who would benefit from a flexible, virtual exercise programme that fits into their daily routines.

As for the structure of SET programmes, compliance with NICE guidance was substantially lower when programme length was considered. The NICE guidance recommends a minimum of 12 weeks of SET, for approximately two hours per week [28]. A previous Cochrane review highlighted that SET programmes that meet the recommended frequency and duration are associated with significant improvements in pain-free and maximum walking distances, whereas less structured or lower-dose interventions show smaller or no such benefits [29].

These findings are consistent with previous literature. A national audit performed before the Covid-19 (between July and December 2019) revealed that only 46% of vascular centres offer SET, with just 6.8% of these centres delivering SET fully compliant with NICE guidelines. Another observational study, conducted after the Covid-19 pandemic, found that only 48% of vascular units had access to SET programmes, with just five out of 23 meeting the NICE dose recommendations [8]. Compared to these earlier reports, our findings suggest a higher proportion of centres are now offering SET, which may indicate some progress in provision. However, there is still substantial room for improvement in aligning practice with NICE recommendations. It should also be noted that this apparent increase needs to be interpreted cautiously, as changes in the total number of vascular units nationally could influence these percentages and may not necessarily reflect an absolute increase in service provision.

However, even if the programme duration is optimal, inadequate programme frequency does not constitute compliance with guidelines. Importantly, international guidelines further stress the need for an adequate exercise dose. The latest ESVS PAD guidelines suggest a training frequency for patients with IC should be three times per week to achieve a meaningful improvement in their walking distance [30]. The results from our FOIR make it difficult to directly compare trends in service provision over time. While some Trusts have achieved the previous benchmark, further improvement is needed to meet the newest guideline recommendations. A similar trend has been identified regarding the provision of other vascular services in NHS England, such as varicose vein interventions, with many local varicose vein commissioning policies not being compliant with NICE guidance [31].

Besides the SET programme dose, it is also important to consider programme attendance and completion rates when thinking about the clinical effectiveness of SET. Although in our FOIR we did not assess SET uptake, attendance, or completion, previous literature indicates that actual uptake among patients with IC is often low, with only 24.2% of eligible patients attending SET [32]. In our previous single-centre observational study, fewer than 20% of patients attended and completed SET [33]. This suggests that even when SET programmes are delivered in accordance with national guidance, poor attendance or high dropout rates may substantially limit their clinical impact.

Compared with previous studies, a large variation in the professional background of staff leading SET was observed in this FOIR. In our study, physiotherapists were the most common group leading classes (25%), whereas in a recent study conducted by Harwood et al., nurses (42%) most commonly led the programmes, followed by physiotherapists (21%) [8]. Regarding the professional background of staff leading IC clinics in our FOIR data, half were nurse-led. Only approximately one in five adopted a multidisciplinary approach, utilising vascular nurse specialists and physiotherapists. This contrasts with cardiac rehabilitation models, which typically feature a cardiac nurse specialist leading a broader multidisciplinary team comprising exercise specialists, dietitians, and a visiting cardiologist [34, 35]. It is important to note that cardiac rehabilitation is traditionally funded by ICBs [36], unlike IC services that are not always funded by the ICB across England. This difference in funding arrangements may help explain the variation in services offered between the two.

Although guidelines recommend a holistic cardiovascular approach, including support for smoking cessation and dietary changes, such services are rarely available to vascular patients in secondary care. In our FOIR, fewer than half (41.5%) of the 82 Trusts offering vascular services reported referring patients to smoking cessation support. While lifestyle interventions are standard in cardiac rehabilitation and are also endorsed by the PAD CQUIN [9], their absence in vascular care highlights a persistent disconnect between clinical guidelines, service delivery, and commissioning. Previous studies have highlighted the burden of PAD diagnosis on wellbeing, such that patients with PAD experience significantly more mental health disorders compared to the general population [37, 38]. Depression in patients with PAD is associated with higher mortality, reduced physical function, and worse outcomes after revascularisation, including major amputation [39]. Despite this, only one of the Trusts that responded to our FOIR reported providing psychological services for patients with PAD. Unfortunately, current care systems for PAD often overlook the integration of mental health support, health behaviour management and the complex nature of living with the disease [40]. These gaps, alongside persistent disparities in PAD care, indicate the need to move beyond the current fragmented care model towards a more integrated, multidisciplinary, patient-focused approach. Further research is essential to develop evidence-based interventions that support the holistic management of patients, as well as tailored, equitable and sustainable care pathways.

### Strengths and limitations

A key strength of this study is that it offers the first comprehensive overview of IC services in England based on publicly available data. The FOIR questionnaire was piloted to ensure its relevance. However, this study has several limitations. Despite our best efforts to obtain responses from all NHS England Trusts and the response rate being high (89.9%), some Trusts did not get back to us, hence, our data is not representative of all NHS England services. However, when compared with the National Vascular Registry (NVR) data on arterial centres in England [41], our FOIR includes information from 61 out of 79 centres within the NVR 2023 update. Moreover, we had a particularly good response from regions with high levels of cardiovascular and peripheral arterial disease, such as the North East coastal regions [42].

Another limitation of FOIR methodology is that the data collected are specific to the questions asked, precluding detailed insights into day-to-day clinical practices in terms of service utilisation or capacity. This FOIR was a descriptive mapping exercise, not an evaluation of service quality or patient outcomes. The undisclosed identity of FOIR responders (either administrative staff or clinicians) within each NHS England Trust introduced potential variability in responses, as answers could be influenced by the responder’s understanding. We did not instruct Trusts to consult clinical teams when completing the FOIR; however, based on our experience and pilot testing, it is standard practice for administrative staff to seek input from clinical colleagues when unsure of the answer. This assumption, while reasonable, cannot be verified and introduces potential variability in internal validity.

To mitigate this, FOIR questions were pilot-tested, and clinical terms were clearly defined. Although attempts were made to seek clarification on some Trust responses, this proved impossible due to the inherent restrictions of the FOIR process, a recognised methodological challenge in such requests [43]. Given the nature of this FOIR, we were unable to access any information on SET uptake, attendance, or completion. This is an important limitation, as programme uptake and completion rates are critical to understanding the real-world effectiveness of SET. Lastly, while this FOIR is focused on NHS England, it is important to note the lack of comparable international literature. There are currently limited published data on the availability and structure of claudication services across other healthcare systems, making direct comparisons difficult. This highlights an urgent need for international research and benchmarking to better understand global variations in PAD care and to guide the development of best practice models.

## Conclusion

The findings reveal regional variation and significant inconsistencies in the provision of IC services across England, with many not meeting guideline recommended provision. The limited availability of specialised claudication services offering medical management, lifestyle interventions and SET is a major obstacle to delivering optimum care for patients with PAD. Only a few Trusts offered comprehensive IC care. Even when SET programmes are available, only a minority of these are delivered in line with national guidance. Understanding the barriers to successful guideline implementation should allow the development of well-considered, effective solutions for patients with PAD. Future research should focus on multidisciplinary integration to ensure equitable and sustainable IC management across the NHS England. Targeted policy interventions, standardised claudication service provision, and strategic investment in vascular care are paramount to improving patient outcomes and aligning clinical practice with established guidelines. 

## Supplementary Information

Below is the link to the electronic supplementary material.

Supplementary Material 1

## Data Availability

The datasets used and/or analysed during the current study are available from the corresponding author on reasonable request.

## Abbreviations

CQUIN: Commissioning for Quality and Innovation
FOIR: Freedom of Information Request
IC: Intermittent Claudication
ICB: Integrated Care Boards
NICE: National Institute for Health and Care Excellence
NHS: National Health System
NVR: National Vascular Registry
PAD: Peripheral Artery Disease
SET: Supervised Exercise Therapy
