# Supplementary material for: Intermittent claudication services in England: insights from a freedom of information request
Source: BMC Health Serv Res. 2026 Feb 23;26:361. doi: 10.1186/s12913-026-14215-9 (PMC12989127; doi:10.1186/s12913-026-14215-9)
Supplement: Supplementary file 1 — Supplementary Material 1 [file 12913_2026_14215_MOESM1_ESM.docx]

**Supplementary Material 1.** Freedom of Information – Claudication Services Questionnaire

**Services**

Q1. Does your Trust currently provide a vascular service for the diagnosis and treatment of vascular diseases, specifically including vascular surgery and interventional vascular radiology?

 Yes

 No

Q2. If vascular services are available, is your organisation designated as a specialist vascular care centre or a hub centre within the network of care providers? (please select all that apply)

***Hub centre****: providing diagnostics and expert advice in an outpatient setting and providing elective and 24/7 emergency vascular services.*

***Spoke centre****: providing diagnostics and expert advice in an outpatient setting.*

 Hub

 Spoke

 Other (Please specify) …………………………………………………………………….

**Claudication clinics**

***Intermittent claudication:*** Intermittent claudication is the most common clinical symptom associated with peripheral arterial disease — walking (exercise) induced pain in the lower limbs caused by diminished circulation that is relieved by rest.

Q3. In your organisation, are there specific claudication clinics for patients with intermittent claudication?

 Yes

 No – If no please skip to question Q10

Q4. If yes, which healthcare professional usually sees patients in these clinics? Please select all that apply.

 Vascular surgeon/medical doctor

 Vascular nurse specialist

 Allied health professional

 Other (Please specify) …………………………………………………………………….

Q5. In your organisation, what is the format of claudication clinics? Please select all that apply.

 Diagnostic testing

 Disease counselling and treatment planning

 Offer lifestyle management

 Offer exercise

 Other (Please specify) ………………………………………………………………..

Q6. How frequently are claudication specialist clinics held?

 Weekly

 Every 2–4 weeks

 Every 2–4 months

 Less frequently than all the above options

 Other (Please specify) ………………………………………………………………..

Q7. What is the standard interval for routine follow-up for patients diagnosed with intermittent claudication?

 Every 1 month,

 Every 3–6 months

 Every 12 months

 Determined by clinical need

 No formal follow-up

 Not Known

 Other (Please specify) …………………………………………………………………….

Q9. In your organisation, what is the current waiting time for patients with intermittent claudication between referral and 1^st^ appointment in the vascular specialist clinics?

Please specify …………………………………………………………………….

**Exercise**

Q10. Are patients with intermittent claudication who attend your Trust offered a supported exercise programme specifically designed for claudication? Please select all that apply.

 Yes

 No

 Verbal advice only

Q11. What is the format of the exercise programme for patients with claudication? Please select all that apply.

 Supervised classes in hospital or community setting

 Virtual Supervised classes at home

 App based directed exercise at home

 Home/Unsupervised

 Don’t Know

Q12. If a structured programme exists, where is this programme provided?

 Your hospital/ Trust, i.e within your organisation

 Another hospital/ Trust, i.e outside your organisation

 Referral to primary care setting outside your organisation

 Referral to a private setting outside your organisation

 Other (Please specify) …………………………………………………………………….

Q13. Over what duration do these supervised exercise programmes typically last for patients diagnosed with claudication?

 4-8 weeks

 12–16 weeks

 More frequently than all the above options (Please specify) …………………..

 Less frequently than all the above options (Please specify) …………………..

Q14. How long are the classes (in minutes)?

 30-60 minutes

 60–90 minutes

 More frequently than all the above options (Please specify) …………………..

 Less frequently than all the above options (Please specify) …………………..

Q15. How often/frequently (times per week) do the classes meet?

 Once per week

 Every 2–4 weeks

 Every 2–4 months

 Less frequently than all the above options

 Other (Please specify) …………………..

Q16. Which healthcare professionals are involved in delivering the classes? Please select all that apply.

 Physiotherapist

 Nurse

 Exercise professional

 Other (Please specify) …………………………………………………………………….

**Smoking**

Q17. In your organisation, where do you offer a smoking cessation service to patients with intermittent claudication?

 Your hospital/ Trust, i.e within your organisation

 Another hospital/ Trust, i.e outside your organisation

 Referral to primary care setting outside your organisation

 Referral to a private setting outside your organisation

 Other (Please specify) …………………………………………………………………….

**Dietetics**

Q18. In your organisation, where do you offer a dietetics service to patients with intermittent claudication?

 Your hospital/ Trust, i.e within your organisation

 Another hospital/ Trust, i.e outside your organisation

 Referral to primary care setting outside your organisation

 Referral to a private setting outside your organisation

 Other (Please specify) …………………………………………………………………….

**Psychology**

Q19. Does your organisation offer a psychology service specific for patients with intermittent claudication?

 Yes

 No – If no please skip to question Q21

Q20. Is there a screening pathway for patients with intermittent claudication to be referred to a psychologist?

 Yes

 No – If no please skip to question Q21

**Referral**

Q21 What are the available pathways or methods by which patients with intermittent claudication are referred to the vascular service in your organisation? Please select all that apply:

 Vascular staff screen lists of hospital ward patients

 A telephone/pager referral system

 A paper referral form (collected in person/faxed/posted)

 Secure email system

 Electronic computerised system (e.g. patient record system)

 Other (Please specify) …………………………………………………………………….

Q22. Is there a standard proforma for referral to vascular?

 Yes

 No

 Other (Please specify) …………………………………………………………………….
